# Herpesvirus reactivation in COVID-19: clinical evidence, mechanistic insights, and research gaps

**DOI:** 10.3389/fimmu.2026.1954424

**Published:** 2026-10-20

**Authors:** Jaeyeong Yoo, Ji Soo Park, Jiyun Kim, Seung-Hyun Lee, Un Yung Choi

**Affiliations:** 1 Department of Internal Medicine, Hallym University Medical Center, Chuncheon Sacred Heart Hospital, Chuncheon, Gangwon, Republic of Korea; 2 Department of Microbiology, Konkuk University School of Medicine, Chungju, Republic of Korea; 3 Department of Infection and Immunology, Konkuk University School of Medicine, Seoul, Republic of Korea; 4 KU Open Innovation Center, Research Institute of Medical Science, Konkuk University School of Medicine, Chungju, Republic of Korea

**Keywords:** herpesvirus latency, herpesvirus reactivation, Long COVID, SARS-CoV-2, viral co-infection

## Abstract

Herpesviruses establish lifelong latency and can reactivate when antiviral immune surveillance is compromised. Since the onset of COVID-19 pandemic, reactivation of multiple human herpesviruses has increasingly been documented, particularly in patients with severe or critical illness. Herpesvirus reactivation has been associated with prolonged intensive care unit stays, secondary infections, greater ventilatory support requirements, and increased mortality. This review summarizes the latency and reactivation characteristics of the major human herpesviruses and evaluates the clinical evidence linking their reactivation to SARS-CoV-2 infection. We further propose a multifactorial mechanistic framework involving impaired T-cell and natural killer cell surveillance, defective interferon-mediated immunity, sustained inflammation and cytokine signaling, direct and indirect effects of SARS-CoV-2 proteins, and host transcriptional and epigenetic reprogramming. However, despite accumulating clinical and experimental evidence supporting a biologically plausible association between SARS-CoV-2 infection and herpesvirus reactivation, the precise mechanisms underlying this interplay remain incompletely understood. Longitudinal viral monitoring, physiologically relevant latency models, and mechanistic intervention studies are therefore needed to establish causality and identify patients who may benefit from targeted surveillance or antiviral treatment.

## 1 Introduction

The emergence of coronavirus disease 2019 (COVID-19), caused by severe acute respiratory syndrome coronavirus 2 (SARS-CoV-2), has had a profound impact on global health, leading to millions of infections and deaths. Beyond its respiratory manifestations, COVID-19 is now recognized as a systemic disease, affecting multiple organ systems and disrupting immune homeostasis (1). A growing body of evidence suggests that SARS-CoV-2 infection induces significant immune dysregulation, including lymphopenia, excessive inflammation, and immune exhaustion, all of which can predispose individuals to opportunistic infections and viral reactivations (2).

Herpesviruses, a large family of double-stranded DNA viruses, establish lifelong latency in their hosts and have the potential to reactivate under conditions of immunosuppression or physiological stress (3). The Herpesviridae family is categorized into three subfamilies: alphaherpesvirinae, betaherpesvirinae, and gammaherpesvirinae (4). Under normal circumstances, the host immune system effectively suppresses these viruses in their latent state. However, various triggers, including immunosuppression, stress, and inflammation, can lead to viral reactivation (5–7).

Since the onset of the COVID-19 pandemic, reports of herpesvirus reactivation in SARS-CoV-2-infected individuals have increased (8), particularly among those with severe disease, prolonged hospitalization, or underlying immune dysfunction (9, 10). Despite these clinical observations, the precise mechanisms linking SARS-CoV-2 infection to herpesvirus reactivation remain incompletely understood. Recent reviews have provided important virus-specific perspectives on the relationship between COVID-19 and herpesvirus latency, including a CMV-centered host–virus resilience framework for aging and Long COVID and a VZV-focused model of post-COVID latency breakdown (11, 12). Building on these perspectives, the present review takes a broader cross-herpesvirus approach encompassing alpha-, beta-, and gammaherpesviruses and integrates evidence from both acute and post-acute COVID-19. Rather than proposing a single unifying causal mechanism, we distinguish direct experimental evidence, indirect mechanistic support, and hypothesis-driven links, while also examining clinical and therapeutic implications and identifying shared and virus-specific research gaps.

In this review, we explore the current evidence on the interplay between COVID-19 and herpesvirus reactivation, discussing the underlying immunological mechanisms, clinical implications, and potential therapeutic strategies. Additionally, we highlight key knowledge gaps and propose future research directions to better understand and manage this viral interplay in the context of emerging infectious diseases.

## 2 Herpesviruses: latency and reactivation

### 2.1 Alphaherpesvirus

Alphaherpesviruses are a subgroup of the Herpesviridae family, characterized by their rapid replication, broad host range, and the establishment of latency in neurons. They replicate rapidly, producing cytopathic effects shortly after infection (13). The major human alpha herpesviruses include herpes simplex virus type 1 (HSV-1), herpes simplex virus type 2 (HSV-2), and varicella-zoster virus (VZV). These viruses primarily establish latency in sensory neuronal ganglia following primary infection and can reactivate when host immune surveillance is compromised (14, 15). Although they share common biological characteristics, they differ in their primary sites of infection, clinical manifestations, and patterns of reactivation (16).

HSV-1 and HSV-2 can infect various anatomical sites, including the lips, oral cavity, and genital region. While HSV-1 is primarily associated with oral herpes and HSV-2 with genital herpes, both viruses can cause infections at either site. The location of latency also differs among these viruses; HSV-1 primarily establishes latency in the trigeminal ganglia, whereas HSV-2 predominantly reside in the sacral ganglia (17). Although HSV-1 latency is classically established in sensory neurons, recent studies have detected the expression of latency-associated transcripts (LATs) in non-neuronal cell types, including dendritic cells and innate lymphoid cells (ILCs) (18). These findings suggest that HSV-1 latency may not be restricted to the trigeminal ganglia, although the biological significance of these alternative reservoirs remains to be clarified.

Following primary varicella (chickenpox), VZV establishes lifelong latency predominantly in the dorsal root and cranial nerve ganglia (19). Similar to HSV, VZV maintains latency through the expression of viral latency-associated transcripts, including the recently identified varicella-zoster virus latency transcript (VLT) (20). Under conditions of aging, immunosuppression, or impaired immune surveillance, latent VZV reactivates and travels along sensory nerves to the skin, causing herpes zoster (shingles) (21). These characteristics make VZV one of the most clinically important alpha herpesviruses associated with aging- and immunosuppression-related viral reactivation.

Despite differences in tissue tropism and clinical manifestations, HSV and VZV share several fundamental mechanisms governing latency and reactivation. Reactivation of alpha herpesviruses occurs when the virus resumes replication within the ganglia and subsequently travels to peripheral tissues, leading to lesions in the skin and mucosa. This reactivation can be triggered by factors such as immunosuppression, stress, and hormonal changes (22). Alpha herpesviruses share common immune evasion strategies. They evade immune surveillance during latency by expressing LAT genes or avoiding detection by immune cells. Additionally, during reactivation, they suppress interferon responses and evade natural killer (NK) cell and T cell responses, allowing them to escape immune control (23).

### 2.2 Betaherpesvirus

Betaherpesvirus is also phylogenetic subfamily of Herpesviridae that includes human cytomegalovirus (CMV or HHV-5), HHV-6A, HHV-6B, HHV-7 (24). Beta-herpesviruses grow slowly in host cells, resulting in comparatively longer reproductive cycles than other HHVs. They also exhibit limited tissue tropism, typically in secretory glands, reticuloendothelial system, especially CD34^+^ myeloid progenitor cells and CD14^+^ monocytes, or kidney cells (25). CMV, named for its ability to induce cytomegaly (abnormal enlargement of cells), persists in various tissues, including kidneys, salivary gland, lymphocytes, macrophages, stem cell, and stromal cell. CMV infection can lead to numerous kinds of complications including pneumonia, retinitis (26). HHV-6 and HHV-7, on the other hand, are featured as roseola infantum (exanthema subitum) causing viruses, hence grouped as roseolovirus. Although they show wide range of tissue tropisms – neural cells, immune cells, epithelium/endothelium, liver, and fibroblasts, they establish latency mainly in CD4^+^ T-lymphocytes or in CD8^+^ T-lymphocytes in some cases for HHV-6A and HHV-7 (27, 28).

Although the factors governing beta herpesvirus reactivation largely overlap with those of alpha herpesviruses, important virus-specific differences exist among members of the betaherpesvirinae. CMV reactivation is most commonly associated with immune dysregulation occurring in conditions such as immunosuppression after transplantation, T-cell deficiency, and sepsis (29–31). Additional triggers include trauma, burns, major surgery, ischemia/reperfusion, and local tissue damage, most of which contribute to a heightened inflammation status (9, 10). At the cellular and molecular level, oxidative stress, DNA damage, differentiation of myeloid cells to dendritic cells or tumor necrosis factor-alpha (TNF-α) are known to be triggers of initiation of lytic cycle of CMV (32). Although much less is known about the reactivation of HHV-6 (6A, 6B) and HHV-7, available evidence suggests that reactivation is mainly associated to immune dysregulation and inflammation. Immunosuppression after transplantation of solid organ or hematopoietic stem cell, by AIDS, or usage of immunosuppressive drugs is often the cause of the reactivation. In other instances, co-infection with Epstein-Barr virus (EBV) or other viruses, physical and psychological stress, hormonal changes from menstruation or pregnancy, medications, or systemic inflammatory response are reported to induce the reactivation (27, 28, 33, 34).

### 2.3 Gammaherpesvirus

Gamma herpesviruses constitute a distinct subfamily within the Herpesviridae family. They are characterized by tropism for lymphoid cells and ability to establish lifelong latency, primarily B lymphocytes. Unlike alpha- and beta-herpesviruses, gamma herpesviruses possess well-established oncogenic potential (35). EBV is associated with a broad spectrum of lymphoid and epithelial malignancies, including Burkitt lymphoma, Hodgkin lymphoma, nasopharyngeal carcinoma, and post-transplant lymphoproliferative disorder (PTLD) (36). KSHV is causally linked to Kaposi’s sarcoma, a vascular tumor frequently observed in immunocompromised individuals, as well as to primary effusion lymphoma (PEL) and multicentric Castleman’s disease (MCD), both of which are rare lymphoproliferative disorders (37).

EBV establishes lifelong latency in memory B cells. After primary infection, the virus enters one of several latency programs. These are categorized as latency 0, I, II, or III, and are defined by selective expression of viral proteins such as Epstein–Barr nuclear antigens (EBNAs) and latent membrane proteins (LMPs), and non-coding RNAs (38, 39). These programs help the virus persist, avoid immune clearance, and occasionally promote lymphomagenesis. Reactivation is triggered by stress signals, inflammatory cytokines, or pharmacological inducers such as HDAC inhibitors. Central to this switch is the immediate-early protein BZLF1, which binds to viral DNA and kickstarts lytic replication (40).

KSHV, similarly, persists in a latent state within B cells, endothelial cells, and fibroblasts. During latency, only a restricted set of viral proteins, including latency-associated nuclear antigen (LANA), viral FLICE inhibitory protein (vFLIP), and vCyclin is expressed (41). Among these, LANA is indispensable for episome maintenance by tethering the viral episome to host chromosomes during cell division (42). In contrast, vFLIP constitutively activates the NF-κB pathway to inhibit apoptosis and promote cell survival (43), whereas vCyclin cooperates with cellular cyclin-dependent kinase 6 (CDK6) to drive cell-cycle progression and support the proliferation of latently infected cells (44). Collectively, these latent proteins facilitate viral persistence by maintaining episomal genomes, promoting host cell survival, and limiting immune-mediated clearance (45). Reactivation hinges on the expression of replication and transcription activator (RTA), a master regulator analogous to EBV’s BZLF1 (46). Like EBV, KSHV reactivation can be provoked by hypoxia, oxidative stress, pro-inflammatory cytokines, or pharmacological agents.

## 3 Clinical evidence of herpesvirus reactivation in COVID-19

### 3.1 HSV and VZV

Accumulating clinical evidence indicates that HSV and VZV, are frequently reactivated during SARS-CoV-2 infection (47). A systematic review and meta-analysis, which included 32 studies published through September 2022, evaluated the cumulative incidence of HHV reactivation in patients with COVID-19. Most of the included studies involved critically ill patients. Among the herpesviruses examined, HSV exhibited one of the highest cumulative incidences of reactivation (38%) (8). HSV reactivation has been documented in a wide spectrum of clinical settings during COVID-19, ranging from recurrent herpes labialis to life-threatening complications such as encephalitis and acute hepatitis. Several studies have reported HSV-1 reactivation rates exceeding 50% among critically ill patients with COVID-19 requiring mechanical ventilation, and HSV reactivation has been associated with prolonged intensive care unit stay and increased mortality (48, 49). More recent ICU studies have further confirmed frequent HSV reactivation in severe COVID-19, with reported rates of approximately 37–48%, although the estimates varied according to sampling methods and diagnostic criteria (50, 51). In addition to mucocutaneous disease, severe complications including HSV-1- and HSV-2-associated encephalitis and acute hepatitis have also been reported in patients with COVID-19 (52, 53).

Reactivation of VZV has likewise been reported throughout the COVID-19 pandemic. Most patients developed classical herpes zoster following SARS-CoV-2 infection, whereas others presented with atypical manifestations, including disseminated varicella-like eruptions (9, 54). Early reports by Recalcati and subsequent multicenter studies by Marzano and colleagues established papulovesicular eruptions as one of the characteristic cutaneous manifestations observed during COVID-19 (55, 56). Although the etiology of these early vesicular eruptions remained uncertain, accumulating clinical evidence published throughout the pandemic has consistently documented herpes zoster following SARS-CoV-2 infection (57, 58). More recently, large retrospective cohort studies from the United States and Spain demonstrated a significantly increased risk of herpes zoster following COVID-19, providing epidemiological support for an association between SARS-CoV-2 infection and VZV reactivation (59, 60). This association was further supported by a 2024 systematic review and meta-analysis of five studies evaluating herpes zoster following COVID-19 infection, which found a 2.16-fold increased risk of herpes zoster among individuals with COVID-19 compared with uninfected controls (61). Together, these findings provide population-level evidence for an association between SARS-CoV-2 infection and VZV reactivation, although they do not establish a direct causal relationship.

### 3.2 CMV, HHV-6, and HHV-7

Substantial clinical evidence also indicates that betaherpesvirus reactivation occurs during COVID-19 (8, 62–64). A recent systematic review and meta-analysis reported pooled cumulative incidences of 19% for CMV, 18% for HHV-6, and 44% for HHV-7 reactivation among patients with COVID-19, indicating that reactivation during COVID-19 has been observed across multiple betaherpesviruses.

Among the betaherpesviruses, CMV has been investigated most extensively. Multiple studies have demonstrated that CMV reactivation occurs predominantly in critically ill patients with COVID-19, particularly those with lymphopenia, prolonged intensive care unit admission, or mechanical ventilation (65, 66). Across these cohorts, CMV reactivation has been associated with prolonged intensive care unit and hospital stays, an increased incidence of secondary bacterial and fungal infections, greater ventilator requirements, and higher mortality (67, 68). Prospective and multicenter cohort studies have further reinforced the association between CMV reactivation and poor clinical outcomes in critically ill patients with COVID-19 (65, 69). More recently, a large longitudinal multi-institutional study of 1,154 hospitalized patients demonstrated CMV reactivation across multiple anatomical compartments using viral transcript detection and linked CMV reactivation to greater COVID-19 severity and clinical outcomes (70). However, whether CMV directly contributes to disease progression or simply reflects profound immune dysfunction remains to be determined.

Clinical evidence for HHV-6 and HHV-7 reactivation is comparatively limited but continues to accumulate. Several reports have described increased frequencies of HHV-6-associated disorders, including pityriasis rosea and Kawasaki-like inflammatory disease, during the COVID-19 pandemic (52, 71). Metagenomic and metatranscriptomic profiling of the gut virome revealed enrichment of betaherpesvirus-associated viral genes in individuals with COVID-19 compared with healthy controls, suggesting that SARS-CoV-2 infection may also be associated with broader alterations in betaherpesvirus-related virome activity (72). Recent study comparing 102 patients with COVID-19 with 51 SARS-CoV-2-negative patients with acute respiratory failure found frequent herpesvirus detection, including HHV-7 in 57% versus 39% of patients, respectively, while HHV-6 was also more frequently detected in the COVID-19 group (73). These findings provide additional clinical evidence for altered HHV-6/7 activity during COVID-19, although viral detection alone does not necessarily indicate clinically meaningful reactivation.

### 3.3 EBV and KSHV

Since the onset of the COVID-19 pandemic, the potential link between SARS-CoV-2 infection and the reactivation of gammaherpesviruses has gained significant attention (74, 75). EBV, a gammaherpesvirus carried by over 90% of the world’s population, is known to reactivate during SARS-CoV-2 infection, particularly in hospitalized patients with moderate to severe disease (10, 76). A systematic review and meta-analysis estimated that approximately 45% of patients with COVID-19 exhibit evidence of EBV reactivation (8), making EBV one of the most commonly reactivated herpesviruses during SARS-CoV-2 infection. Several studies further reported that EBV reactivation is associated with elevated inflammatory markers, including IL-6, prolonged hospitalization, intensive care unit admission, and increased disease severity (75). Although these observations do not establish a causal relationship, they consistently suggest that EBV reactivation accompanies profound immune dysregulation during severe COVID-19.

Beyond acute COVID-19, EBV reactivation has been proposed as a potential contributor to post-acute sequelae of SARS-CoV-2 infection (PASC), commonly referred to as Long COVID. Serological evidence of reactivation was detected in 66.7% of individuals with persistent symptoms, compared with 10% of recovered controls, and EBV early antigen-diffuse IgG levels correlated with the overall symptom burden (77). In parallel, EBV viremia was detectable in 14% of patients at the time of initial COVID-19 diagnosis and was associated with the subsequent development of PASC (78). These findings suggest that EBV reactivation may occur early during SARS-CoV-2 infection and be associated with an increased risk of persistent symptoms such as fatigue, insomnia, headache, myalgia, and cognitive impairment, rather than arising solely as a consequence of prolonged illness. However, the relationship between EBV reactivation and Long COVID remains unresolved. Altmann et al. found no significant differences in SARS-CoV-2-specific antibody or T-cell responses between individuals with persistent symptoms and those who fully recovered after mild or asymptomatic infection (79). They also observed no difference in T-cell responses to a peptide pool containing EBV, CMV, and influenza epitopes, although this approach did not directly measure EBV DNAemia or conventional serological markers of EBV reactivation. Differences in disease severity, sampling time, study population, and the criteria used to define EBV reactivation may therefore account for some of the discrepancies among studies. Current evidence supports EBV reactivation as a potential biomarker or contributing factor in a subset of patients with Long COVID, but does not yet establish a direct causal role.

In contrast, clinical evidence for KSHV reactivation during COVID-19 remains relatively limited (80). Initial observations were based on isolated case reports, including the recurrence of cutaneous Kaposi’s sarcoma in a patient whose skin biopsy contained particles interpreted as KSHV and SARS-CoV-2 by transmission electron microscopy (81), and the development of disseminated Kaposi’s sarcoma in a 61-year-old HIV-negative man following severe COVID-19 (82). These cases raised the possibility that SARS-CoV-2-associated immune dysregulation, together with immunosuppressive treatment, may facilitate KSHV reactivation or Kaposi’s sarcoma progression. Supportive observational evidence from South African patients further showed associations between detectable KSHV viral load and SARS-CoV-2 exposure or antibody levels among unvaccinated adults with HIV, as well as between KSHV viral load and mortality among hospitalized patients with COVID-19 (83). Although these findings extend the evidence beyond individual case reports, they do not establish whether SARS-CoV-2 directly promotes KSHV reactivation, whether KSHV contributes to adverse COVID-19 outcomes, or whether both reflect shared immune dysfunction.

The biological characteristics and clinical evidence of major human herpesvirus reactivation during COVID-19 are summarized in **Table 1**.

**Table 1:** Biological characteristics and clinical evidence of herpesvirus reactivation on COVID-19.

Herpesvirus Subfamily	Specific Virus (HHV)	Biological Latency Site	Clinical Reactivation Manifestations	Study / Reported Reactivation Frequency or Risk in COVID-19	Methodological Context	Clinical Outcomes and Associations	Key References
Alpha	Varicella-zoster virus (VZV)	‣ Sensory neurons in dorsal root ganglia ‣ Cranial nerve ganglia	‣ Herpes zoster (shingles) ‣ Disseminated varicella-like eruptions ‣ Papulovesicular eruptions	‣ Wang et al., 2024 (61) 2.16-fold higher risk of herpes zoster after COVID-19 vs uninfected controls (95% CI, 1.24–3.76)	‣ Systematic review/meta-analysis of 5 studies ‣ Clinical HZ occurrence/recurrence rather than protocolized virologic surveillance ‣ Not directly comparable with PCR-based HHV reactivation frequencies	‣ Increased HZ risk after COVID-19 ‣ Impaired immune surveillance and Th17–IL-17 signaling proposed as contributing mechanisms	Biology: [13, 17–20] Clinical: [57] Mechanism: [99]
	HHV-1 / HHV-2	‣ Sensory neuronal ganglia ‣ Trigeminal ganglia (HSV-1) ‣ Sacral ganglia (HSV-2) ‣ HSV-1 LATs also reported in dendritic cells and ILCs	‣ Herpes labialis ‣ Genital herpes ‣ Encephalitis ‣ Acute hepatitis	‣ Shafiee et al., 2023 (8) 38% pooled cumulative incidence (95% CI, 28–50%; I²=86%)	‣ Systematic review/meta-analysis ‣ Predominantly severe/critical COVID-19 ‣ PCR-based definitions; substantial heterogeneity	‣ Associated with prolonged ICU stay and mortality ‣ Type I IFN-neutralizing autoantibodies may increase reactivation risk	Biology: [12,14–16,20,21] Clinical: [45–49] Immune association: [91]
				‣ Mattei et al., 2024 (50) 48% HSV-1; 2.5% HSV-2	‣ 120 critically ill patients ‣ Blood PCR at ICU admission and weekly thereafter		
				‣ Boers et al., 2024 (51) 37% pulmonary HSV-1 reactivation	‣ Mechanically ventilated, HSV-seropositive patients ‣ BAL-based definition of pulmonary reactivation		
				‣ Ravanini et al., 2025 (73) 27% HSV-1 overall; 44% in severe/critical COVID-19	‣ Mixed-severity cohort ‣ Nasopharyngeal qPCR ‣ 2% in SARS-CoV-2-negative respiratory-failure controls		
				‣ Michel et al., 2026 (84) ~24% any HSV-1 detection/reactivation	‣ 455 consecutive ICU patients ‣ Whole-blood and respiratory-sample screening ‣ Includes isolated HSV-1 and HSV-1/CMV co-reactivation		
Beta	Cytomegalovirus (CMV/HHV-5)	‣ CD34+ myeloid progenitors ‣ Monocytes/macrophages ‣ Kidneys and salivary glands ‣ Stromal cells	‣ Pneumonia ‣ Retinitis ‣ Often subclinical DNAemia/viremia	‣ Shafiee et al., 2023 (8) 19% pooled cumulative incidence (95% CI, 13–28%; I²=87%)	‣ Systematic review/meta-analysis ‣ Mostly severe/critical cohorts ‣ High between-study heterogeneity	‣ Prolonged ICU/hospital stay ‣ Secondary bacterial/fungal infections ‣ Greater ventilatory requirements ‣ Increased mortality	Biology: [23,24,27] Clinical: [45,57,60–64] Mechanism: [96]
				‣ Tassaneeyasin et al., 2024 (68) 28.1% CMV reactivation	‣ 185 critically ill patients with COVID-19 pneumonia ‣ Quantitative CMV PCR screening ‣ Higher cumulative corticosteroid exposure associated with reactivation		
				‣ Boers et al., 2024 (51) 6% pulmonary CMV reactivation	‣ Mechanically ventilated, CMV-seropositive patients ‣ BAL-based pulmonary reactivation definition		
				‣ Ravanini et al., 2025 (73) 8% overall; 22% in severe/critical COVID-19	‣ Mixed-severity cohort ‣ Nasopharyngeal qPCR ‣ 4% in SARS-CoV-2-negative respiratory-failure controls		
				‣ Michel et al., 2026 (84) ~24% any CMV detection/reactivation	‣ 455 consecutive ICU patients ‣ Whole-blood and respiratory-sample screening ‣ Includes isolated CMV and HSV-1/CMV co-reactivation		
	HHV-6 (HHV-6A and HHV-6B)	‣ Predominantly CD4+ T cells ‣ Also reported in CD8+ T cells and neural, endothelial, epithelial, hepatic, and fibroblast compartments	‣ Roseola infantum ‣ Pityriasis rosea / Kawasaki-like inflammatory presentations reported during COVID-19 ‣ Reactivation often subclinical	‣ Shafiee et al., 2023 (8) 18% pooled cumulative incidence (95% CI, 8–35%)	‣ Pooled estimate based on relatively few studies ‣ Definitions and sampling strategies varied	‣ More often detected in severe/critical COVID-19 ‣ Virus-specific clinical significance remains uncertain	Biology: [25,30,31] Clinical: [45,48,58,59,65] Recent: Ravanini 2025
				‣ Ravanini et al., 2025 (73) 46% overall; 56% in severe/critical COVID-19	‣ Nasopharyngeal qPCR ‣ 22% positivity in SARS-CoV-2-negative respiratory-failure controls ‣ Respiratory DNA positivity ≠ systemic DNAemia		
	HHV-7	‣ Predominantly CD4+ T cells ‣ Broader immune and neural tissue tropism	‣ Exanthema subitum (roseola) ‣ Reactivation commonly subclinical and detected by molecular surveillance	‣ Shafiee et al., 2023 (8) 44% pooled cumulative incidence (95% CI, 32–56%)	‣ Systematic review/meta-analysis ‣ Limited virus-specific clinical data	‣ Detected in severe COVID-19 cohorts ‣ Virus-specific clinical consequences remain poorly defined	Biology: [26,31] Clinical: [45,59] Recent: Ravanini 2025
				‣ Ravanini et al., 2025 (73) 57% overall; 72% in severe/critical COVID-19	‣ Nasopharyngeal qPCR ‣ 39% positivity in SARS-CoV-2-negative respiratory-failure controls ‣ High background detection limits COVID-19 specificity		
Gamma	Kaposi's sarcoma-associated herpesvirus (KSHV/HHV-8)	‣ B cells ‣ Endothelial cells ‣ Fibroblasts	‣ Lytic reactivation ‣ Cutaneous/disseminated Kaposi's sarcoma ‣ Primary effusion lymphoma ‣ Multicentric Castleman's disease	‣ Shafiee et al., 2023 (8) 19% pooled cumulative incidence (95% CI, 14–26%)	‣ Pooled estimate based on limited HHV-8/KSHV data	‣ Clinical evidence remains limited ‣ KSHV viral load associated with SARS-CoV-2 exposure and mortality in selected cohorts ‣ SARS-CoV-2 proteins/live-virus co-infection can promote lytic activation in vitro	Biology: [34,38–43] Incidence: [45] Clinical: [72–75] Mechanism: [102,103]
				‣ Lambarey et al., 2024 (83) 21% had detectable peripheral-blood KSHV viral load overall Quarterly detection: 3.3% → 69.2%	‣ Non-hospitalized HIV-positive cohort ‣ CD4 < 350 cells/µL ‣ Peripheral-blood KSHV viral load used as reactivation proxy ‣ Not generalizable to the broader COVID-19 population		
	Epstein–Barr virus (EBV)	‣ Memory B cells	‣ EBV DNAemia / lytic reactivation ‣ Possible association with Long COVID symptoms ‣ Lymphoproliferative and epithelial malignancies	‣ Shafiee et al., 2023 (8) 45% pooled cumulative incidence (95% CI, 28–63%; I²=96%)	‣ Systematic review/meta-analysis ‣ Very high heterogeneity across studies	‣ Associated with elevated IL-6 and greater acute COVID-19 severity ‣ Possible association with PASC/Long COVID; causality unresolved	Biology: [33,35–37,85,86] Acute COVID-19: [10,45,67,68] Long COVID: [69–71] Mechanism: [98,104]
				‣ Mattei et al., 2024 (50) 65% EBV reactivation	‣ 120 critically ill patients ‣ Blood PCR at ICU admission and weekly thereafter		
				‣ Ravanini et al., 2025 (73) 39% overall; 47% in severe/critical COVID-19	‣ Mixed-severity cohort ‣ Nasopharyngeal qPCR ‣ 18% in SARS-CoV-2-negative respiratory-failure controls		
				‣ Maguire et al., 2026 (70) 24% with detectable EBV transcripts near admission (days 1–8)	‣ 1,154 hospitalized patients across 20 U.S. hospitals ‣ Longitudinal RNA-seq across PBMC, nasal, and endotracheal compartments ‣ Viral-transcript detection differs from DNAemia/serology		

Methodological note: ‣ Pooled meta-analytic estimates and single-cohort frequencies are not directly interchangeable. ‣ Reported rates vary with disease severity, specimen type, assay (DNA qPCR vs viral RNA-seq), sampling frequency, serostatus eligibility, and reactivation thresholds. ‣ Recent cohort values are therefore presented as contextual evidence rather than replacements for pooled estimates.

## 4 Proposed mechanisms underlying herpesvirus reactivation in COVID-19

Although herpesvirus reactivation has been repeatedly documented in patients with COVID-19, mechanistic studies directly linking SARS-CoV-2 infection to the disruption of herpesvirus latency remain limited. The evidence discussed below therefore varies in strength. Direct experimental evidence refers to studies in which SARS-CoV-2 infection, SARS-CoV-2-derived factors, or patient-derived samples were experimentally shown to promote herpesvirus lytic activation. Indirect experimental evidence includes studies demonstrating that immune, inflammatory, or cellular stress pathways altered during COVID-19 can induce herpesvirus reactivation in other experimental settings. In contrast, hypothesis-driven mechanisms are biologically plausible connections inferred from parallel observations but not yet directly demonstrated in the context of SARS-CoV-2 infection. Together, these findings support a multifactorial model involving impaired antiviral immune surveillance, inflammatory signaling, viral interactions, cellular stress, and transcriptional or epigenetic remodeling.

### 4.1 Impaired antiviral immune surveillance

The mechanistic link between COVID-19-associated lymphocyte dysfunction and herpesvirus reactivation is supported primarily by indirect evidence rather than by direct experimental demonstration. Lymphopenia is a prominent feature of severe COVID-19 and frequently involves reductions in circulating CD4^+^ and CD8^+^ T cells and natural NK cells (85–87). The remaining lymphocytes may also exhibit impaired proliferation, cytokine production, degranulation, and cytotoxicity, together with increased expression of exhaustion- or senescence-associated markers such as PD-1, TIM-3, NKG2A, and CD57 (88, 89). Reduced CD8^+^ T-cell and NK-Cell activity may allow reactivating herpesvirus infected cells to escape immune clearance, as demonstrated in experimental model of EBV infection (90, 91). Consistent with this possibility, a recent study of patients with moderate-to-critical COVID-19 found EBV reactivation to be associated with an altered CD8+ T-cell phenotype, including reduced CD28 and CD11a expression and increased CD57 expression (92). However, the cross-sectional design did not establish whether T-cell dysfunction preceded or resulted from EBV reactivation (92). Severe COVID-19 is also associated with a reduction in circulating memory B cells and disruption of long-term humoral immunity (93, 94). This may weaken virus-specific antibody responses and disturb the coordination between humoral and cellular immunity. Because memory B cells are the principal reservoir of latent EBV (95, 96), changes in their abundance, activation, or differentiation may additionally affect the balance between EBV latency and lytic reactivation (**Figure 1**). However, the relevance of memory B-cell loss to other herpesviruses is less clearly established.

**Figure 1:**
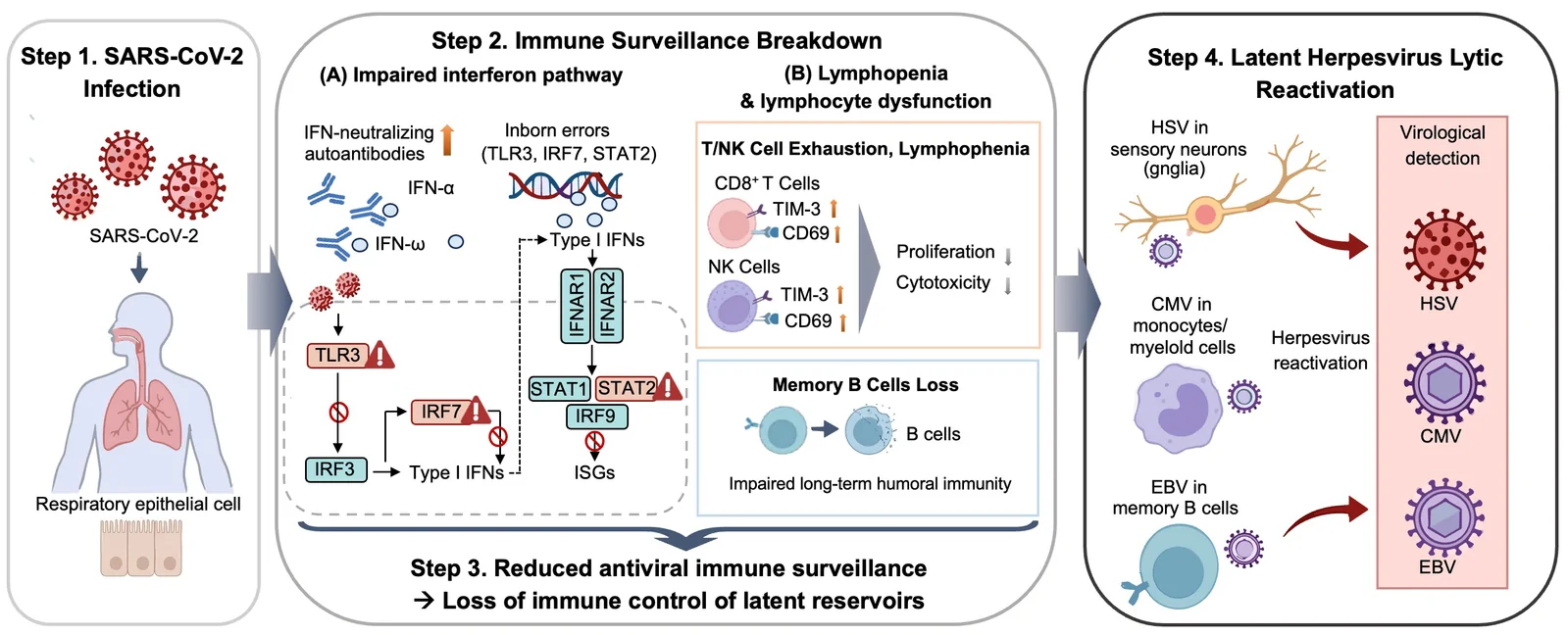
Impaired antiviral immune surveillance promotes herpesvirus reactivation during SARS-CoV-2 infection. SARS-CoV-2 infection may disrupt immune mechanisms required to maintain herpesvirus latency through two major pathways. First, impaired type I interferon signaling may result from neutralizing autoantibodies against type I interferons or inborn errors affecting viral sensing, interferon production, or downstream IFNAR–STAT signaling, thereby reducing interferon-stimulated gene expression and antiviral activity. Second, COVID-19-associated lymphopenia and functional impairment of CD8+ T cells and NK cells, together with alterations in the memory B-cell compartment, may weaken cellular and humoral immune surveillance. These defects may collectively reduce immune control of latent herpesvirus reservoirs, including sensory neurons for HSV, monocytes and myeloid cells for CMV, and memory B cells for EBV, thereby facilitating lytic reactivation and subsequent clinical or virological detection. Created in BioRender. Choi, U. Y. (2026) https://BioRender.com/rpt47uo.

A similar hypothesis is supported by clinical and genetic evidence implicating impaired interferon-mediated immunity, although a direct causal pathway from SARS-CoV-2-induced interferon dysfunction to herpesvirus reactivation has not been experimentally established. Inborn errors affecting TLR3, IRF7 or STAT2 can disrupt type I and type III interferon responses and the expression of interferon-stimulated genes (97, 98). Defects in these pathways have been associated with severe viral infections and, particularly in the TLR3–interferon axis, with susceptibility to herpes simplex encephalitis (99, 100). Thus, SARS-CoV-2 and herpesviruses may exploit overlapping defects in host antiviral defense (**Figure 1**). Accordingly, it is plausible that HSV-associated diseases may occur concurrently with severe COVID-19, particularly in patients with underlying impairment of interferon-mediated immunity. Neutralizing autoantibodies against type I interferons provide a comparable acquired defect. Autoantibodies targeting IFN-α and IFN-ω have been detected in approximately 10% of patients with life-threatening COVID-19 and have also been identified in respiratory secretions. By suppressing local and systemic interferon activity, these autoantibodies may weaken mucosal antiviral defense and increase the risk of herpesvirus reactivation (**Figure 1**). A significant association has been reported between the presence of these autoantibodies and CMV or HSV reactivation, although it remains unclear whether they directly promote reactivation or primarily indicate profound antiviral immune dysfunction (101).

### 4.2 Hyperinflammation and cytokine-driven reactivation

In addition to impaired antiviral immune surveillance, the hyperinflammatory environment associated with COVID-19 may provide biochemical signals that favor herpesvirus reactivation. Clinical evidence from patients with severe COVID-19 demonstrates sustained production of inflammatory mediators, including IL-6, IL-1β, TNF-α, IL-8, IL-10, and TGF-β (102, 103). Prolonged cytokine exposure may also impair antiviral effector functions (104). In particular, elevated IL-6 and TGF-β have been associated with reduced cytotoxicity, degranulation, and cytokine production by CD8^+^ T cells and NK cells, creating a paradoxical state in which systemic inflammation coexists with weakened antiviral immunity (87, 105). Experimental studies in established herpesvirus latency models further show that inflammatory cytokines can directly promote viral lytic activation. For example, TNF-α induced immediate-early viral gene expression and the production of infectious virus in latently HCMV-infected cells through pathways involving NF-κB, KAP1, and ATM activation (32). In latently infected neurons, IL-1β-induced neuronal hyperexcitability activated the DLK–JNK stress pathway and initiated HSV-1 lytic gene expression (106). These findings demonstrate that inflammatory signals can promote herpesvirus reactivation in a virus- and cell-type-specific manner, although these experimental studies were not conducted in the setting of SARS-CoV-2 infection.

More direct support for cytokine-driven reactivation has emerged from mechanistic studies using patient-derived samples in multisystem inflammatory syndrome in children. Elevated TGF-β was associated with impaired virus-reactive T-cell responses and reduced monocyte antigen presentation, while patient serum induced EBV lytic reactivation *in vitro*. Blocking TGF-β reversed both the immune dysfunction and EBV-reactivating activity, supporting a mechanistic connection between cytokine dysregulation, impaired immune surveillance, and herpesvirus reactivation (107).

By contrast, the proposed involvement of the Th17–IL-17 axis in VZV reactivation remains hypothesis-generating rather than experimentally established. The Th17–IL-17 axis has been proposed as an additional link between COVID-19-associated inflammation and VZV reactivation. A bioinformatic comparison of COVID-19 and herpes zoster identified convergence on Th17 differentiation and IL-17 signaling, together with broader inflammatory cytokine pathways involving mediators such as TNF and IL-6 described above (108). These shared genes were primarily enriched in cytokine–cytokine receptor interaction and JAK–STAT signaling pathways. The combined protein-interaction network further revealed enhanced connectivity among genes involved in chemokine signaling, antigen presentation, immune-cell recruitment, and inflammatory tissue responses. These findings suggest that amplification of the Th17–IL-17 axis within a broader inflammatory network may generate an environment favorable to VZV reactivation. However, because this evidence is based on database-derived gene associations, it does not establish that IL-17A directly induces VZV replication or reactivation.

### 4.3 Direct and indirect interaction between SARS-CoV-2 and herpesviruses

Experimental findings showing that SARS-CoV-2-encoded protein can activate inflammatory signaling provide indirect support for their potential role in herpesvirus reactivation. Secreted ORF8 can stimulate monocytes and macrophages to produce inflammatory cytokines. Although one study attributed this response to ORF8 binding to the IL-17 receptor complex and activating downstream NF-κB signaling, a subsequent study found that ORF8-induced inflammatory signaling was dependent on MyD88 but independent of the IL-17 receptor (109, 110). Thus, ORF8-mediated paracrine signaling may alter the inflammatory environment surrounding herpesvirus reservoirs and indirectly favor reactivation, although ORF8 has not been shown to directly induce herpesviral lytic replication.

More direct experimental evidence has been obtained for KSHV. Ectopic expression of the SARS-CoV-2 spike or nucleocapsid protein in KSHV-infected iSLK.219 and primary effusion lymphoma cells enhanced viral lytic gene expression and increased the production of infectious virions (111). Extending these findings, a subsequent study established a live-virus co-infection model in which KSHV-infected A549-hACE2 cells were infected with replication-competent SARS-CoV-2. Co-infection induced the KSHV immediate-early gene RTA, early gene ORF59, and late gene ORF17 in a time- and dose-dependent manner, providing direct evidence that live SARS-CoV-2 infection can promote KSHV lytic activation in cells permissive to both viruses (112). Transcriptomic analysis further revealed broad alterations in inflammatory, mitochondrial, metabolic, and stress-responsive pathways, suggesting that SARS-CoV-2 may facilitate KSHV reactivation through both viral protein-mediated effects and extensive remodeling of the host cellular environment. However, the causal contribution of these host pathways remains to be experimentally validated.

A potential bidirectional interaction between SARS-CoV-2 and herpesviruses has also been demonstrated in experimental models. In EBV-infected epithelial cells, lytic reactivation markedly increased ACE2 expression and enhanced ACE2-dependent entry of SARS-CoV-2 spike-pseudotyped virus. This effect was mediated in part by the EBV transactivator Zta, which activated the methylated ACE2 promoter (113). These findings suggest that EBV reactivation may increase epithelial susceptibility to SARS-CoV-2 entry, although this has not yet been confirmed using authentic SARS-CoV-2 or *in vivo* models.

### 4.4 Host transcriptional and epigenetic remodeling

The proposed link between SARS-CoV-2-induced epigenetic remodeling and herpesvirus reactivation is currently largely hypothesis-driven. Experimental evidence demonstrates that SARS-CoV-2 infection reorganizes the host three-dimensional genome, weakens chromatin contacts, alters histone modifications, suppresses interferon-response genes, and increases the activating mark H3K4me3 at pro-inflammatory gene promoters (114). Furthermore, severe COVID-19 can also establish persistent epigenetic programs in monocytes and hematopoietic stem and progenitor cells that remain detectable for months to one year after recovery (115). Such acute and long-lasting epigenetic reprogramming may contribute to delayed or persistent impairment of herpesvirus control. In addition, although the subcellular localization and chromatin-associated function of ORF8 remain debated (116), ORF8 has been reported to act as a histone H3 mimic that associates with chromatin and interferes with histone post-translational modifications (117).

On the herpesvirus side, epigenetic regulation of viral latency and reactivation is well established. Herpesvirus latency is maintained through chromatinization of viral episomes, with DNA methylation and repressive histone modifications restricting lytic-gene transcription; reactivation requires virus-specific mechanisms that remodel or overcome this epigenetic repression (118). Given these established features of SARS-CoV-2 infection and herpesvirus latency, it is plausible to hypothesize that SARS-CoV-2-induced disruption of host chromatin regulation could lower the threshold for herpesviral lytic activation, although direct remodeling of latent herpesviral episomes by SARS-CoV-2 has not yet been demonstrated.

Experimental evidence from KSHV models shows that hypoxia can promote lytic reactivation, whereas its proposed contribution to KSHV reactivation during COVID-19 remains hypothetical. Hypoxemia and tissue hypoxia are common features of acute respiratory distress syndrome in severe COVID-19 (119). In KSHV-infected cells, hypoxia can induce expression of the viral RTA through HIF-1α-associated transcriptional signaling, thereby promoting lytic reactivation (120). Although this mechanism has not been directly demonstrated in patients with COVID-19, severe disease-associated hypoxia may create conditions permissive for KSHV reactivation.

## 5 Clinical and therapeutic implications and future directions

### 5.1 Breakdown of immune tolerance and autoimmune consequences

Numerous studies reported higher incidence rate of autoimmune disease or inflammatory disease in COVID-19, and some important cohort studies have shown there has been statistically important increment in incidence of the numerous diseases, namely, rheumatoid arthritis (RA), systemic lupus erythematosus (SLE), multisystem inflammatory syndrome (MIS), and IgA vasculitis (121–125). Even though the arousal of autoimmune response in COVID-19 infection is thought to be mainly caused by immune dysregulation during infection, a few recent studies have proposed possible linkages among COVID-19, autoimmune consequences, and herpesviruses.

It is well established that EBV infection is a strong risk factor for multiple sclerosis (126). Given that EBV reactivation is prominently exacerbated during COVID-19, it is highly plausible that similar immune-dysregulating mechanisms elicit a burst of autoimmune responses. Furthermore, since EBV viremia serves as a strong predictive biomarker for long COVID, a reciprocal or mutually amplifying relationship is indicated (78). Recent studies provide direct molecular and cellular evidence supporting this axis. HHV-6 reactivation has been associated with autoimmunity against tight junction proteins, including zonulin and occludin, and with increased SARS-CoV-2 IgM levels (127), while HHV-6-associated exhaustion of NK cells may further impair immune surveillance and create the immunological prerequisites for subsequent autoimmune processes (128). This association is also supported by strong correlation between endogenous HHV-6B positivity and higher SLE disease activity, together with high anellovirus load, a surrogate marker for impaired immune surveillance, with SLE, RA, and COVID-19 (129). Collectively, these studies are implicating that SARS-CoV-2 infection induced immune dysregulation may facilitate HHV-6 reactivation, which could reinforce barrier dysfunction, impaired immune surveillance, and autoimmune responses.

However, it is noteworthy that Long COVID may not universally manifest as classic systemic autoimmune diseases. A recent cohort study evaluating SARS-CoV-2 and herpesvirus co-infections found no distinct antinuclear autoantibody profiles differentiating Long COVID patients from fully recovered controls (130). This suggests that the herpesvirus-driven breakdown of immune tolerance in COVID-19 is likely characterized by localized immunopathology or broader humoral dysregulation, rather than conventional high-titer systemic autoimmunity.

### 5.2 Therapeutic strategies in COVID-19-related herpes reactivations

Although immunosuppression is a well-known risk factor for herpesvirus reactivation (131), clinical outcomes regarding treatment strategies on COVID-19-related herpes reactivation have shown somewhat paradoxical results. Antivirals such as acyclovir or ganciclovir have been used in selected patients with documented herpesvirus reactivation, although evidence supporting their routine use in COVID-19 remains limited (132–134). However, corticosteroids are also heavily utilized to mitigate the severe hyperinflammatory response in COVID-19. Interestingly, several studies have reported that corticosteroid use and lymphopenia are not statistically associated with an increased risk of herpes reactivation (63, 65). A case report by Lu Y et al. (135) even demonstrated successful clinical outcomes using an immunosuppressive strategy in severely ill COVID-19 patients with concurrent herpes reactivation. Furthermore, given recent evidence that remdesivir treatment for COVID-19 can induce the reactivation of EBV and KSHV at the molecular level (136), it is plausible that COVID-19-associated herpes reactivation may also be driven by direct cellular stress and intracellular interactions in addition to systemic immunosuppression.

Consequently, there are growing demands to consider prophylactic antiviral therapies upon COVID-19 diagnosis or prior to initiating immunomodulatory treatments. A few studies have shown that the herpesviral load during acute COVID-19 infection significantly influences overall treatment outcomes and mortality (51, 84, 137). As recent findings suggest that long COVID could be driven by herpesvirus reactivation during the acute phase of infection (138) – for instance, EBV or CMV reactivation is considered a culprit for the neurologic manifestations of long COVID (47)– severely ill patients or those in the PASC could potentially benefit from targeted prophylactic treatment with established antivirals (139).

### 5.3 Future research directions in COVID-19-associated herpesvirus reactivation

A major priority is to establish more consistent clinical and epidemiological evidence for herpesvirus reactivation during COVID-19. Current studies differ substantially in patient populations, sampling schedules, anatomical compartments, diagnostic assays, and criteria used to define reactivation, limiting direct comparisons across studies. Standardized definitions for herpesvirus infection or disease, including CMV, EBV, HSV, and VZV, have been developed in other clinical settings, particularly transplantation and hematologic disease (140–142), but comparable criteria remain lacking for COVID-19-associated herpesvirus reactivation. Future longitudinal cohorts should therefore adopt standardized, virus-specific definitions that distinguish viral detection or DNAemia from biologically or clinically meaningful reactivation. Serial assessment of viral DNA, lytic transcripts, serological markers, virus-specific immunity, and clinical symptoms will further help clarify the temporal relationship between herpesvirus reactivation and COVID-19 outcomes.

A second priority is the development of physiologically relevant experimental models that more faithfully reproduce herpesvirus latency and reactivation. Many current mechanistic studies rely on transformed cell lines, viral-protein overexpression, or pseudotyped viruses, which are useful for identifying candidate pathways but do not fully recapitulate latent infection in its native cellular or tissue context. Reservoir- and tissue-relevant models are increasingly being developed to better recapitulate herpesvirus latency and reactivation, including primary or stem-cell-derived cells, organoids and other three-dimensional systems, ex vivo models, and humanized or other *in vivo* models (143–145). These platforms should be further integrated with authentic infection and co-infection systems to determine whether SARS-CoV-2-associated immune, inflammatory, metabolic, or epigenetic alterations directly perturb herpesvirus latency and reactivation.

A separate challenge is to determine how these findings should be translated into clinical practice. Current evidence does not support universal herpesvirus screening in all patients with COVID-19. Targeted surveillance may be more appropriate for patients at higher risk of herpesvirus reactivation, particularly those with critical illness, prolonged mechanical ventilation, marked lymphopenia or other evidence of impaired antiviral immunity, and clinical findings suggestive of herpesvirus disease (49, 52, 62, 101). Prospective intervention studies are needed to determine whether virus-specific monitoring or antiviral treatment improves clinically meaningful outcomes and to identify which patient populations are most likely to benefit.

## 6 Conclusion and discussion

Accumulating evidence indicates that herpesvirus reactivation is a clinically relevant but heterogeneous feature of COVID-19, particularly in patients with severe or critical illness (8, 64). However, it remains uncertain whether reactivation directly contributes to disease progression or primarily reflects profound immune dysfunction. The available findings support a multifactorial model involving impaired antiviral immune surveillance, defective interferon responses, sustained inflammatory signaling, direct or indirect effects of SARS-CoV-2 proteins, and host transcriptional and epigenetic remodeling. These mechanisms may act together to disturb the balance between herpesviral latency and lytic replication.

The relationship between herpesvirus reactivation and Long COVID remains particularly important but should be interpreted primarily as an association rather than as evidence of causality. Among the herpesviruses, EBV has been studied most extensively. Several observational studies have reported higher frequencies of EBV reactivation markers in individuals with persistent post-COVID-19 symptoms (77, 146), and EBV viremia during acute SARS-CoV-2 infection has been associated with the subsequent development of PASC (78). However, these findings have not been consistent across cohorts. One study found no difference in T-cell responses to a peptide pool containing EBV epitopes between individuals with persistent symptoms and recovered controls (79), while a recent large longitudinal study found no higher rate of acute EBV transcription among patients who subsequently developed Long COVID (70). These discrepancies may reflect differences in disease severity, sampling time, study populations, and, importantly, the markers used to define viral reactivation. Evidence for other herpesviruses is even more limited. A recent multicohort study found that early HHV-6-specific IgA responses were enriched among individuals who subsequently developed Long COVID, but these serological findings indicate altered herpesvirus-directed immune responses rather than demonstrating systemic HHV-6 reactivation (147). Longer-term evidence for KSHV is restricted largely to individual cases; in one two-year follow-up study, persistent KSHV viremia preceded SARS-CoV-2 infection, making a causal contribution of SARS-CoV-2 difficult to establish (148).

One possible explanation for the observed long-term association between COVID-19 and herpesvirus reactivation is that SARS-CoV-2 infection may leave persistent biological alterations that extend beyond the acute phase. Longitudinal studies have demonstrated sustained abnormalities in innate and adaptive immunity, including persistent inflammatory signaling and alterations in T- and B-cell compartments for months after infection (149). In addition, SARS-CoV-2 RNA or antigen can persist in multiple tissues after clinical recovery, potentially providing continued antigenic or inflammatory stimulation (150). Severe COVID-19 can also induce durable epigenetic reprogramming of hematopoietic stem and progenitor cells and their myeloid progeny that remains detectable for up to one year (115), while persistent immune–epithelial interactions and impaired tissue repair may create chronically altered local microenvironments after viral pneumonia (151). Together, these long-lasting host alterations provide a biologically plausible basis for prolonged disruption of herpesvirus latency, although their direct contribution to delayed reactivation remains to be established.

The lifelong persistence of herpesviruses means that immune disruption caused by an emerging infection may have consequences extending beyond the acute phase (3, 4, 47). Future pandemics may similarly destabilize latent infections through immune, inflammatory, or cellular stress pathways. Continued integration of long-term clinical surveillance with physiologically relevant mechanistic models will therefore be essential to determine whether herpesvirus reactivation is principally a biomarker of severe disease, a modifiable contributor to acute and post-acute complications, or a combination of both.

### 6.1 Literature search and selection strategy

Relevant literature was identified primarily through PubMed, with Google Scholar used as a supplementary search tool. For studies examining the relationship between SARS-CoV-2 infection and herpesvirus reactivation, we focused mainly on publications from 2020 onward using combinations of terms related to COVID-19 or SARS-CoV-2, herpesvirus reactivation, and individual herpesviruses, including HSV, VZV, CMV, HHV-6, HHV-7, EBV, and KSHV. Original clinical studies, mechanistic studies, systematic reviews, and meta-analyses were prioritized according to their relevance to the topics addressed in this review. Reference lists of key articles and reviews were also examined to identify additional relevant studies. For established mechanisms of herpesvirus latency and reactivation, earlier landmark and highly cited studies were included when necessary. Mechanistic studies were selected based on their biological relevance to immune, inflammatory, cellular stress, or transcriptional changes observed during COVID-19, while the strength of evidence was considered when distinguishing directly demonstrated mechanisms from indirect or hypothesis-generating links.
